# Chemical Control of Snail Vectors as an Integrated Part of a Strategy for the Elimination of Schistosomiasis—A Review of the State of Knowledge and Future Needs

**DOI:** 10.3390/tropicalmed9090222

**Published:** 2024-09-20

**Authors:** Amadou Garba Djirmay, Rajpal Singh Yadav, Jiagang Guo, David Rollinson, Henry Madsen

**Affiliations:** 1 Department of Control of Neglected Tropical Diseases, World Health Organization, 1211 Geneva 27, Switzerland; rajpal@yadav.cloud (R.S.Y.);; 2 Academy of Public Health Entomology, Udaipur 313002, India; 3 Global Schistosomiasis Alliance, Ealing Cross, 85 Uxbridge Road, Ealing, London W5 5BW, UK; 4 Wolfson Wellcome Biomedical Laboratories, Science Department, Natural History Museum, Cromwell Road, London SW7 5BD, UK; 5 Department of Veterinary and Animal Sciences, Faculty of Health and Medical Sciences, University of Copenhagen, Dyrlaegevej 100, 1870 Frederiksberg C, Denmark

**Keywords:** schistosomiasis, snails, molluscicides, chemical snail control, environmental management, health education, community, water supply and sanitation, behavioural change

## Abstract

WHO promotes the implementation of a comprehensive strategy to control and eliminate schistosomiasis through preventive chemotherapy, snail control, clean water supply, improved sanitation, behaviour change interventions, and environmental management. The transmission of schistosomiasis involves infected definitive hosts (humans or animals) excreting eggs that hatch (miracidia), which infect freshwater snail vectors (also referred to as intermediate snail hosts) living in marshlands, ponds, lakes, rivers, or irrigation canals. Infective larvae (cercariae) develop within the snail, which, when released, may infect humans and/or animals in contact with the water. Snail control aims to interrupt the transmission cycle of the disease by removing the vector snails and, by so doing, indirectly improves the impact of the preventive chemotherapy by reducing reinfection. Snail control was, for many years, the only strategy for the prevention of schistosomiasis before preventive chemotherapy became the primary intervention. Snails can be controlled through various methods: environmental control, biological control, and chemical control. The chemical control of snails has proven to be the most effective method to interrupt the transmission of schistosomiasis. The current review aims to describe the vector snails of human schistosomiasis, present the chemicals and strategies for the control of snails, the challenges with the implementation, and the future needs. Snail control can play a key role in reducing schistosomiasis transmission and, thus, complements other interventions for disease control. There is a need to develop new molluscicide products or new formulations and methods of applications for existing molluscicides that would target snail vectors more specifically.

## 1. Introduction

Schistosomiasis or bilharzias is a major neglected tropical disease, with more than 200 million people requiring treatment and close to 800 million at risk [1]. The burden of the disease is estimated to exceed 1.8million disability-adjusted life-years in 2021 [2]. Schistosomiasis is caused by trematodes of the genus *Schistosoma*. The transmission cycle requires the contamination of surface water by excreta, specific species of freshwater snails as intermediate hosts (snail vectors), and human–water contact [3]. The disease is endemic in many countries in Africa, the Middle East, South America, and Asia. WHO estimates that 78 countries are endemic for schistosomiasis globally [4].

Each of the schistosome species has its characteristic spectrum of snail vectors [5]. The main disease-causing species are *S. haematobium*, *S. mansoni*, and *S. japonicum*. In addition, some species—*S. intercalatum*, *S. guineensis*, and *S. mekongi*—have more limited geographical distribution, and some other schistosome species primarily infect animals [5]. Hybridization between human species such as *S. haematobium* and *S. guineensis*, and also between human and animal species has been documented in Africa [6,7]. The intermediate hosts of *S. mansoni* belong to the Planorbidae and those of *S. haematobium* and *S. intercalatum* belong to the family Bulinidae [8,9], while those of *S. japonicum* and *S. mekongi* belong to the family Pomatiopsidae [10].

Schistosomiasis is transmitted by skin contact with infected water and manifests in genitourinary (*S. haematobium*) and intestinal clinical forms (other schistosomes). Penetration of the skin by cercariae can result in urticaria, pruritus, cough, malaise, etc., called cercarial dermatitis [1]. Typical urogenital signs are blood in the urine with frequent genital signs, in particular in women such as dyspareunia, dysmenorrhea, leucorrhoea, menstrual disorders, and post-coital bleeding, while intestinal signs are diarrhoea, blood in the stool, or abdominal pain. Chronic infection results in fibrosis of the bladder and ureter, which may progress to hydronephrosis in urogenital schistosomiasis, liver fibrosis, and portal hypertension with ascites and haematemesis [1,3,4] in the intestinal form.

The current WHO strategy for controlling and eliminating schistosomiasis depends on an integration of preventive chemotherapy, health education, behavioural change interventions, snail control, environmental management, and improved water supply and sanitation.

The availability of a safe, effective, and easy-to-administer medicine, praziquantel, which is efficacious against all schistosome species, previously made morbidity control through the large-scale treatment of populations at risk as the primary strategy for the control of schistosomiasis [11]. Before the advent of praziquantel, the main global strategy to fight schistosomiasis relied on snail control as recommended by a WHO expert committee in 1960. The expert committee report emphasized that of the several control approaches available at that time, snail control appeared to be the most feasible and cost-effective [12].

The launch of the neglected tropical disease (NTD) roadmap in 2012 and the implementation of large-scale preventive chemotherapy programmes led to the treatment of millions of at-risk people worldwide with high coverage, in particular in school-aged children, the age group with the highest intensity of infection [13]. In 2017 alone, more than a hundred million people received preventive chemotherapy for schistosomiasis. This resulted in a drastic global reduction in the infection prevalence and intensities [14,15,16,17,18,19] such that interruption of disease transmission could be envisaged. However, preventive chemotherapy alone cannot eliminate the disease. The use of integrated approaches including transmission control measures is required [20,21].

The WHO Global Vector Control Response 2017–2030 [22] has set a goal of reducing at least 40% of the incidence of vector-borne disease, including schistosomiasis by 2025.

WHO recommends focal mollusciciding in areas where disease elimination is the goal and in transmission hot spots, which are areas where, despite repeated mass drug administration, the prevalence of the disease remains high [23,24].

The aim of this review is to detail the snails involved in schistosomiasis transmission and to review the snail control strategies with an emphasis on the use of molluscicides.

## 2. Intermediate Host Snails

**Figure 1:**
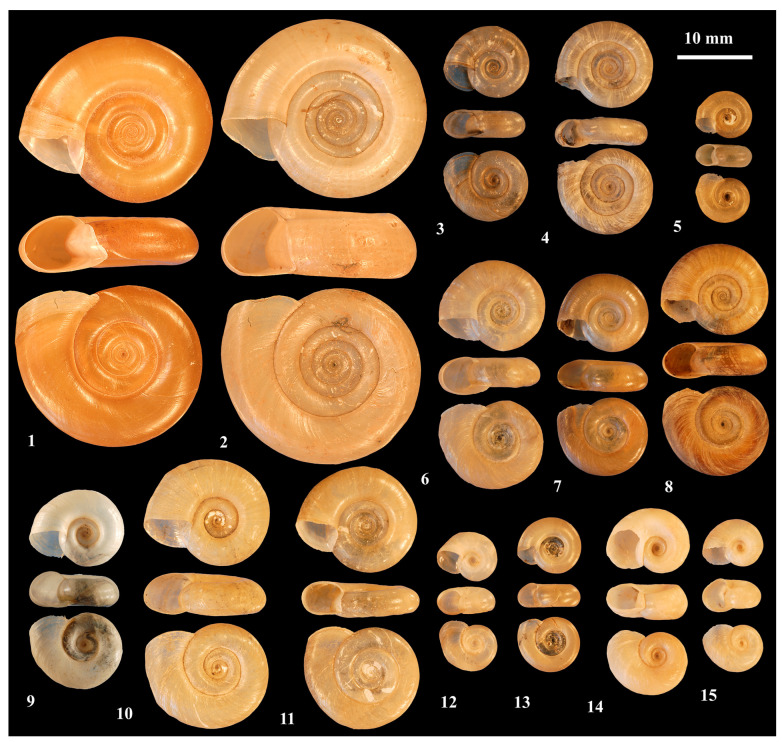
Neotropical (1–5) and African (6–15) *Biomphalaria* species, (1) *B. glabrata*; (2) *B. tenagophila*; (3) *B. straminea*; (4) *B. havanensis*; (5) *B. helophila*; (6) *B. alexandrina*; (7) *B. angulosa*; (8) *B. camerunensis*; (9) *B. pfeifferi*; (10) *B. salinarum*; (11) *B. sudanica*; (12) *B. choanomphala*; (13) *B. rhodesiensis* (14) *B. smithi*; and (15) *B. stanleyi* (prepared by HM).

**Figure 2:**
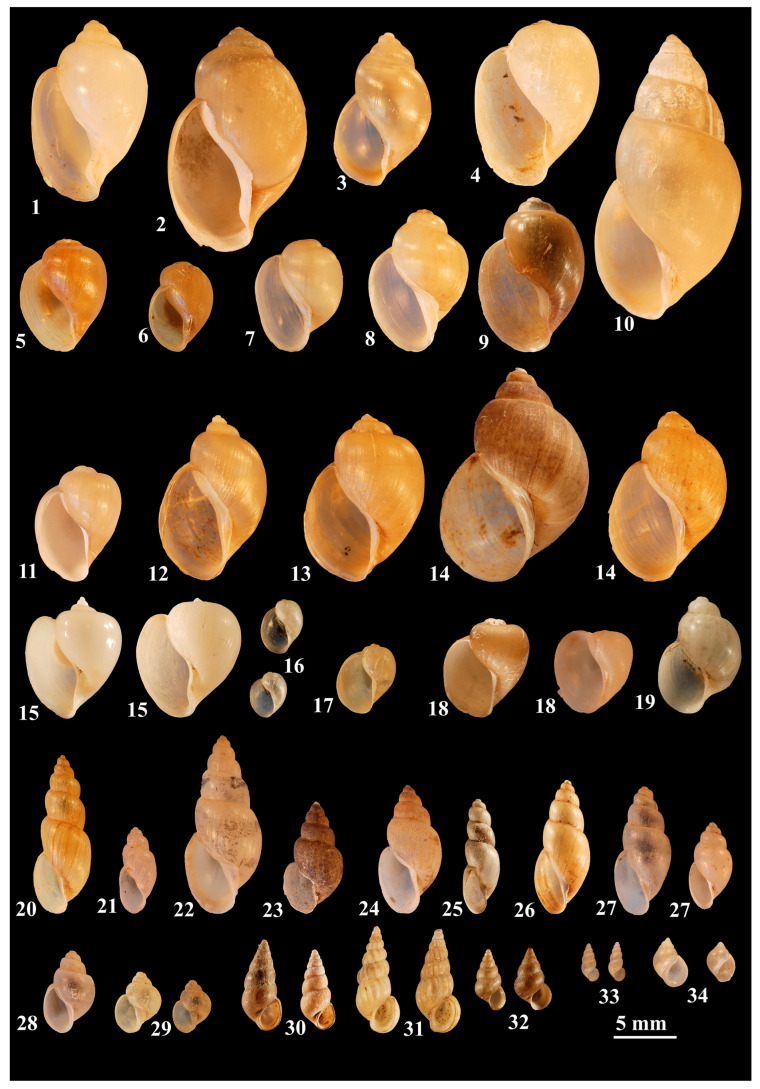
Representative species of the *Bulinus* species groups, *Oncomelania*, *Robertsiella*, and *Neotricula* species. **The *B. africanus* group:** (1) *B. abyssinicus*; (2) *B. africanus*; (3) *B. nasutus*; (4) *B. ugandae*; (5) *B. jousseaumei*; (6) *B. obtusus*; (7) *B. obtusispira*; (8) *B. umbilicatus*; (9) *B. globosus*; (10) *B. productus.* ***B. truncatus/tropicus* complex:** (11) *B. angolensis*; (12) *B. liratus*; (13) *B. natalensis*; (14) *B. tropicus*; (15) *B*. *nyassanus*; (16) *B. succinoides*; (17) *B. transversalis*; (18) *B. trigonus*; (19) *B. truncatus*. **The *B. forskalii* group:** (20) *B. bavayi*; (21) *B. beccarii*; (22) *B. canescens*; (23) *B. cernicus*; (24) *B. crystallinus*; (25) *B. forskalii*; (26) *B. scalaris*; (27) *B. senegalensis*. **The *B. reticulatus* group:** (28) *B. reticulatus*; (29) *B. wrighti*. **Asian species:** *Oncomelania hupensis*. (30) smooth form and (31) ribbed form; (32) *O. quadrasi*; (33) *Neotricula aperta*; (34) *Robertsiella kaporensis* (prepared by HM).

Various species of *Biomphalaria* spp. (Figure 1) serve as intermediate hosts for *S. mansoni*, and certain *Bulinus* species (Figure 2) are intermediate hosts for *S. haematobium* and *S. intercalatum*. Certain subspecies of *Oncomelania hupensis* (Figure 2) are known to transmit *S. japonicum* [23]. Some of these subspecies, however, should be recognized as full species, i.e., *O. quadrasi* [24,25] and *O. lindoensis* [26]. *Neotricula aperta* (Figure 2) transmits *S. mekongi* and *Robertsiella kaporensis* (Figure 2) *S. malayensis* [10].

*Biomphalaria* spp. and *Bulinus* spp. are aquatic and hermaphroditic snails that can be found in almost all types of water bodies, ranging from small temporary ponds or streams to large lakes and rivers [8]. Artificial water bodies such as irrigation canals and dams are particularly suitable habitats that, because of the intense human–water contact, may play a very important role in the transmission of schistosomiasis. Most species of these genera are usually found in shallow waters and often in association with aquatic macrophytes; however, in some of the large lakes, they may be found also at considerable depth, for example, *Biomphalaria* spp. in the East African lakes and *Bulinus nyassanus* in Lake Malawi [27]. *Schistosoma*-infected snails, however, are primarily found in more shallow waters (depth < 2 m). Snails may be widely distributed in an area, but *Schistosoma*-infected snails are focally distributed due to the focality of human–water contacts. Generally, the prevalence of infection with schistosomes in snails is low (1–5%) and yet prevalence in people can be very high as seen in lake-shore villages at Lake Malawi [28,29]; however, the focal prevalence of infection in snails may be quite high, e.g., up to 30% in Mali [30]. In large irrigation schemes, most infected *Biomphalaria* spp. and *Bulinus* spp. snails are found close to human settlements [31]. Focality and seasonality in the transmission of schistosomes are key issues that need to be considered for snail control.

The eggs of *Biomphalaria* spp. and *Bulinus* spp. are deposited in masses on plants or solid objects in the water. Under optimal temperature conditions, hatching takes place after 7–10 days. Snails may reach sexual maturity after 3–15 weeks [32]. The average longevity varies among species and with local conditions. Their reproductive capacity is high, and many of these species are capable of both “outcrossing or selfing” and, hence, one snail can generate a new population. Seasonally, these species may undergo marked fluctuations in density depending on rainfall and/or temperature as the main causative factors.

Snails of *Oncomelania* spp. are small, amphibious, and dioecious. Females tend to be larger than males. Eggs are laid singly on solid objects. Hatching occurs after 10–25 days, depending on temperature, and newly hatched snails pass through an aquatic stage of 1–2 weeks. Snails reach sexual maturity after 10–16 weeks and may live for 24–35 weeks [33]. Their reproductive potential is low compared with that of pulmonate snails, and recolonization of sites treated with molluscicide may be slow [33].

*Oncomelania* spp. inhabit flood plains, especially artificial habitats, resulting from agricultural development, drainage channels, roadside ditches, and rice fields; small canals and drainage canals of irrigation works are important [33,34,35]. Snails are found primarily on the banks of flood plains but some are also found in very shallow waters (i.e., depths less than c. 20 cm). Habitats preferred by *O. hupensis* are shaded by vegetation, and the temperature is relatively constant and cool. Water currents of above 0.14 m/s are generally unfavourable for *O. hupensis*.

*Neotricula aperta* snails transmit *S. mekongi*, which primarily infects humans across the central Lao People’s Democratic Republic and eastern Cambodia [10]. Three strains of *N. aperta* have been identified on the basis of body size and mantle pigmentation [36]. All three strains are suitable hosts for *S. mekongi*; however, in nature, only the γ-strain is known to be epidemiologically significant [37]. *N. aperta* is found in large rivers in Central Laos, Thailand, and eastern Cambodia but there is also a spring-dwelling form of the species [10]. Population densities seem to be controlled by the annual rise and fall in the water level of the river. During the rainy season, the flow is torrential, and the species seems to persist as eggs attach to the underside of stones. Female snails live less than a year and apparently lay large numbers of eggs before the onset of the rains.

In Malaysia, *S. malayensis* infects mainly rodents but can also infect humans; the intermediate hosts are species of *Robertsiella* (Pomatiopsidae: Triculinae) [38,39]. *R. kaporensis* may be found in small streams attached to rocks, leaves, and twigs [10]. Moreover, *R. silvicola* lives in seepages and springs [39].

## 3. Approaches to Snail Control

Snails can be controlled using molluscicides (synthetic or of natural origin), environmental modification, and biological control; however, chemical snail control yields rapid reductions in snail density and disease transmission. To have an impact, snail control activities need to be sustained in the long term. Distribution of the intermediate hosts is governed by a multitude of ecological factors, some of which are more important than others. Detailed information on such factors would be useful in combination with data on human settlements and water contact activities to predict high-risk areas for transmission and for focusing mollusciciding efforts [40].

### 3.1. History of Molluscicides in Schistosomiasis Control

The use of molluscicides in Asia started at the beginning of the 20th century in Japan [41]. The potential value of inorganic molluscicides in controlling African schistosomiasis was noted as far back as 1915 when Leiper suggested that snail control in irrigation canals in Egypt could be improved by using ammonium sulfate in pools remaining after the canals had been emptied [42]. In 1942, the Egyptian Ministry of Health established a Snail Destruction Section in Fayoum [43]. Copper sulfate was the molluscicide of choice against the target snail *Bulinus truncatus* [43]. Serious attempts to evaluate candidate molluscicidal compounds did not begin, however, until the late 1940s to 1950s, when about 7000 compounds were screened for mollusciciding efficacy [44]. Interest in molluscicides peaked in the 1960s but declined during the 1970s due partly to the exceedingly high costs of developing new compounds and increasing concern for environmental impacts [43].

The control of snails was initially considered to be the main intervention against schistosomiasis and was attempted through the large-scale use of snail toxins (molluscicides), partly because safe drugs for treating infected people were not available. However, when praziquantel became available, the role of snail control changed to become a supportive measure but was still the “recommended” approach in schistosomiasis control programmes in the late 1970s [45]. By the 1990s, there was increasing concern about the ethical and environmental consequences of large-scale molluscicide spraying operations. Obviously, extensive use of molluscicides could have severe effects on biodiversity as well [46,47], and this could damage food chains. Snails play a key role in freshwater ecosystems as primary consumers and secondary producers forming the food basis for many other taxa. Although the disease may be widespread, the transmission of schistosomes is focal, i.e., not all water contact sites are transmission sites, and, hence, the application of molluscicides should be restricted to such areas [31,48,49].

The effectiveness of chemical control of snails on transmission of schistosomiasis has been demonstrated by various reviews [50,51,52]. A new WHO manual on mollusciciding recommends the use of focal mollusciciding when the elimination of schistosomiasis is the goal of the programme in transmission hot spots or in case of emergencies [13].

### 3.2. Chemical Molluscicides

The potential of available molluscicides in snail control has been reviewed by several authors over time [53,54,55,56,57]. Although the list of molluscicides commercially available at present can be reduced to just one compound, namely, niclosamide (Bayluscide or Mollutox) [58]. Some of the most important compounds used previously include lime, copper sulfate and other copper compounds [59,60,61], trifenmorph [59,60,62], sodium pentachlorophenate (NaPCP)) [55,63] organotin and related compounds [64,65], nicontinanilide, and others. Sodium 2,5-dichloro-4-bromophenol, named B2, has shown promising efficacy against *O. nosophora* [66,67] and *O. hupensis* [68]. The B2 compound was tested against aquatic snails in South Africa and had potential activity, but Bayluscide remained the molluscicide of choice for snail control [69].

Fluoracetamide, bromoacetamide, and chloroacetamide have shown molluscicidal activity against amphibious snails [70,71,72,73]. These compounds show low toxicity to fish and may be suitable for application in fishponds. Metaldehyde is used extensively to control terrestrial snails [74] and its activity against amphibious and aquatic snails has also been tested [75,76]. It appears that metaldehyde is unique among molluscicides in producing degradation products such as acetate, which attracts both aquatic snails and slugs such as *Derocerus reticulatum* [77].

For the control of terrestrial snails, compounds are often formulated as baits, and there is special interest in iron-phosphate, which is efficient against terrestrial snails in bait formulations [78,79,80]. It can even be used in organic farms where other molluscicides are banned [78]. Its use against apple snails, *Pomacea canaliculata*, has been attempted, and although the response (increased mortality) was delayed, the compound (sold as “Ferroxx AQ”) should be further tested against freshwater snails [81].

In freshwater aquaculture ponds, lime is often applied but often not in a form that can be used for snail control. Hydrated lime sprayed in catfish ponds along the banks, however, is efficient in controlling snails, serving as intermediate hosts for *Bolbophorus* spp. [82,83,84]. In a review of chemicals used in aquaculture in Asia [85], there is mention of some organotin-based molluscicides being used; however, these are now banned. Furthermore, in some areas, tobacco dust or leaves may have molluscicidal activity when used in aquaculture ponds.

### 3.3. Plant-Based Molluscicides

Given the high costs of imported synthetic molluscicides, the use of plants with molluscicidal activity in snail control is an attractive approach [86,87,88]. A great many plants have been evaluated for their molluscicidal activity [89]. These plants could be cultivated locally and applied by local people. The potential of plant-origin molluscicides was first recognized in 1933 when Archibald observed the toxicity to *Biomphalaria sudanica* of the berries of the tree *Balanites aegyptiaca* in Sudan [43]. The first paper on *Phytolacca dodecandra* or endod, still the most extensively studied plant molluscicide, was published by Lemma [90]. Unfortunately, the extract of this plant has no toxicity to snail eggs. Some studies have looked at essential oils isolated from plants [91,92]; spraying crop oils on egg masses of *Pomacea maculata* greatly reduced the hatching rate [93].

Currently, there is an increasing interest in compounds from marine algae, especially cyanobacteria (blue-green algae), which are known to produce potent toxins [94,95,96,97,98,99]. Benthic marine cyanobacteria are known for their prolific biosynthetic capacities to produce structurally diverse secondary metabolites with biomedical application and their ability to form harmful cyanobacterial algal blooms [100]. Toxins from *Pseudomonas fluorescens* proved highly selective for control of the invasive zebra mussel (*Dreissena polymorpha*), which causes major problems in the cooling systems of power stations [101]. The product was developed into a commercial product, Zequanox^®^, which is now used routinely for the control of zebra and quagga mussels [101]. The product is composed of dead microorganisms and has no acute mammalian toxicity [102]. Laboratory exposure of *Biomphalaria alexandrina* to Phycocyanin derived from three cyanobacteria, *Anabaena oryzae*, *Nostoc muscorum*, and *Spirulina platensis*, showed promising molluscicidal activity, while the compounds were reported to be safe to Tilapia fish as the survival rate was 100% at the effective molluscicidal concentrations [103]. Further studies on testing toxins from such sources against schistosome intermediate hosts should be encouraged.

*Lantana camara*, used as dusting powder at 0.06% concentration, has shown good molluscicidal activity and preservation of fish (*Letidocephalus guntea*) in a study conducted in Bangladesh [104], showing a potential use of this invasive plant.

Plant and other bio-derived molluscicides should undergo the same detailed toxicological, mutagenic, and carcinogenic testing as synthetic molluscicides; although of natural origin, they are still chemicals [13,105]. Many of these plants have been used by the local people as fish poisons [106].

### 3.4. Methods of Molluscicide Application

**Figure 3:**
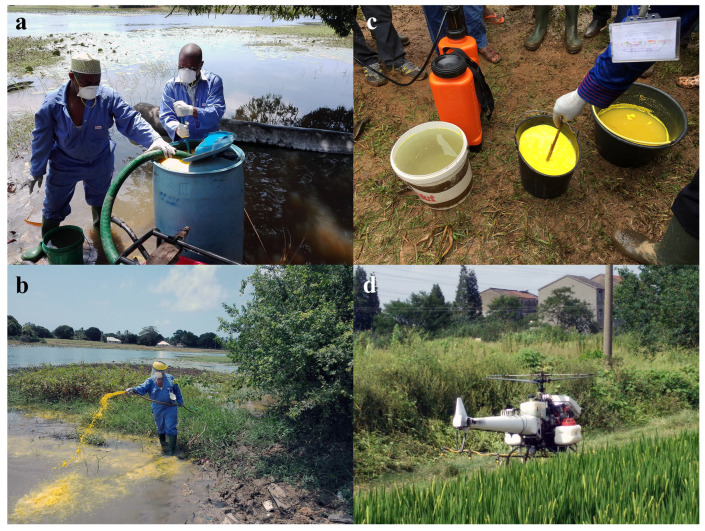
(**a**) Preparation for molluscicide application in Zanzibar; (**b**) Molluscicide application in a temporary pond in Zanzibar; (**c**) Preparation of niclosamide for application in Cameroon; (**d**) Molluscicide application in China using a drone ((**a**–**c**): AGD; (**d**): courtesy NIPD, China).

During the 1960s and 1970s, molluscicides were applied on a large scale in multiple ways (Figure 3) (drip-feeding, spraying using different systems, aerial applications) using many different compounds and formulations (solutions, wettable powders, granule formulations, slow-release formulations, and baits). An overview of past strategies for molluscicide application is given in Table 1. Diverse types of water bodies demand different treatment strategies.

Focal application of molluscicides in transmission sites only may require higher dosage because water movement (wave action or current) will quickly dilute the chemical. Focal or partial treatments in large rivers or lakes may not be possible unless the transmission site is isolated from the main body of water. Such a strategy was tried in Lake Volta [107].

Focal control of snails requires the identification of all transmission sites; however, the absence of infected snails, as judged by cercarial shedding, from a given site is a poor indicator of the absence of transmission because the prevalence of infection in snails may be very low. The use of polymerase chain reaction techniques to detect pre-patent infections in snails improves identification of transmission sites [108,109,110] as would the use of environmental DNA to detect schistosome cercariae in water, but this will add expenses [111]. If a focal application at transmission sites only is employed, it should be kept in mind that cercariae are not necessarily produced within human–water contact sites, as cercariae can be transported some distance with water flow. This could be an issue, particularly for the control of *S. japonicum*. Dense beds of submerged aquatic macrophytes may act as a barrier to penetration of the chemical as the exposure time in the focal application is relatively short [112].

There may be special consideration for controlling *Oncomelania* spp., and this is partly because of its amphibious life. Many snails may be found outside the water, for example, attached to vegetation. Thus, in China, to improve the effectiveness of mollusciciding, vegetation (mainly grasses) is cut down before application. Mollusciciding strategies generally include immersion, spraying, and slow-release methods, and mollusciciding is often combined with environmental modification.

## 4. Molluscicide Formulations

A widening of the range of available auxiliary surface-active agents (emulsifiers, spreaders, stickers, wetters, dispersants), naturally occurring and synthetic fillers, stabilizers (e.g., antioxidants and water scavengers), and solvents combined with the accumulation of considerable knowledge about their use have made possible considerable advances in the formulation of pesticides for agricultural purposes and for the control of disease vectors or intermediate host snails. It is, therefore, generally possible, though not necessarily easy, to formulate active ingredients in such a way that the resulting molluscicide formulations are stable and have an acceptable storage life in the tropics.

Various granule formulations (floating or sinking) have been attempted for the delivery of molluscicides in places otherwise difficult to treat [113,114,115,116]. For example, Bayluscide suspension was used to soak corncobs and treat dense stands of stout hydrophytes where spraying was not possible [117] or where capsules (mixture of sand, molluscicide, and binder) were applied that would sink to the bottom of deeper lentic habitats and release the chemical there [118].

Slow-release formulations using different matrices and different molluscicides have been used with some success [43,54,119]. Tributyltin oxide (TBTO), the most efficient formulation [120,121,122], was used extensively in anti-fouling paints; however, it is responsible for imposex in snails and other taxa as well (for example, female snails developing male organs), which is a common phenomenon in areas around harbours. Imposex has also been reported in some freshwater snails [123]. Hence, the compound has been banned in many places. Other slow-release formulations include copper compounds in different matrices [124,125,126,127]. Several studies have used gelatin granules containing attractants to get snails into the vicinity of the granules slowly releasing the molluscicide [128,129].

Bait formulations have been tried with some success against different snail species [130,131]; it was found that attractants are species (or genus) specific and actually induce the devouring of the bait as well as cause mortality. There has been limited follow-up on this idea for schistosomiasis control except for the work by Thomas and co-workers (reviewed in [132]). Bait formulations, however, are widely used for the control of terrestrial snails (slugs and snails) in agriculture or horticulture [133], and there have been several studies using bait formulations against lymnaeid species [134], *Indoplanorbis exustus* [135], and *Pomacea maculata* [129].

New formulations of existing molluscicides might improve their molluscicidal effect. Thus, in China, a new formulation, a 25% suspension concentrate of niclosamide, was found to have better molluscicidal effects against *O. hupensis* than the conventional 50% wettable powder formulation [136]. Niclosamide is also available as a new 4% wettable powder developed in China [137]. The suspension concentrate formulation was also found to be active against *Biomphalaria glabrata*, the intermediate host of *S. mansoni* [138]. Another promising formulation is a 26% suspension concentrate of metaldehyde and niclosamide [139].

Recently, a metaldehyde and niclosamide 26% suspension concentrate formulation has been developed [76]. Its 24 h LC_50_ value is estimated to be 0.0583 mg/L against *O. hupensis* snails, and its field trials showed 97.5–100% snail mortalities following immersion in 2 g AI/m^3^ for 24, 48, and 72 h [76].

Recently, there has been interest in applying nanotechnology to develop molluscicide formulations. Thus, certain compounds formulated as nanoparticles that show promise as molluscicides have been developed [140,141,142]. This approach should be studied further.

## 5. Market for Molluscicides

The market share of public health pesticides including molluscicides is very small compared with that of agricultural pesticides. There are many challenges in developing and marketing new molluscicides. The market for molluscicides to control schistosome snail vectors may not be large enough for chemical companies to take a renewed interest in developing and registering new molluscicides, and public health budgets may not prioritize the development and use of new molluscicides. The costs of registering molluscicides are high and the expected revenue seems to be low.

There are, however, many situations in which molluscicides could be used to control snails as intermediate hosts of schistosomiasis and other diseases caused by trematodes (swimmer’s itch due to avian schistosomes, fascioliasis, opisthorchiasis, clonorchiasis, intestinal trematodiases) or as agricultural pests, for example, apple snails (primarily *P. canaliculata*) in Asia. Molluscicides may also be used to control other invasive mollusc species, for example, the bivalve *Dreissena polymorpha* [143,144,145]. Since most molluscicides, including niclosamide, also have piscicidal effects, some may be employed to control invasive fish species. Thus, a granular formulation of Bayluscide has been used either to estimate the density of larvae of the invasive sea lamprey (*Petromyzon marinus*) or to control them in lentic habitats [146].

The reduction in schistosomiasis prevalence and low compliance of the population for preventive chemotherapy [147] could be reasons for an increase in molluscicide usage. The WHA65.21 resolution calls for endemic countries to take full advantage of non-health programmes to improve the environment in order to cut the transmission of schistosomiasis and accelerate its elimination. In addition, the expected benefit for the tourism industry of WHO recognition of countries as free of schistosomiasis may be a further driver of molluscicide usage.

The newly issued guidelines for the evaluations of molluscicides [148] will encourage manufacturers to apply for the WHO prequalification of their products. If successful, this will increase the availability of molluscicides and decrease their cost due to market competition.

Some of the above formulations are commercially available but would need to be properly evaluated for their safety, human and environmental risks, as well as efficacy in snail control and registered according to the guidelines of the Organisation for Economic Co-operation and Development and national regulations before they can be exported and used elsewhere [149].

## 6. Development of New Molluscicides

In searching for new molluscicides, products should be identified that possess as many of the following characteristics of an ideal molluscicide as possible.

- Toxic to all life stages of the snails (i.e., egg masses, juvenile and adult snails).
- Remain effective in water at low concentrations for a prolonged period.
- Should not repel snails.
- Neither acutely or chronically toxic to humans and domestic animals nor to aquatic life in general.
- Non-toxic to irrigated crops and should not lead to toxic residues in such crops.
- Should be in a form or formulation that is easy and safe to handle, apply, and readily dispersible in water.
- Should be stable in storage during the shelf-life period.
- Should be easily and cheaply transported and can be applied at low operational costs.
- Have an affordable price.

Of the list of molluscicides produced so far, niclosamide seems to be the one most closely approaching the ideal molluscicide; however, there are issues with its wider toxicity.

The use of broad-spectrum molluscicides to control the snail hosts of schistosomes is problematic; therefore, the focus should be on compounds or formulations targeting intermediate hosts as selectively as possible. This is mainly due to environmental concerns about pesticide pollution in general [150] and the costs of large-scale mollusciciding operations.

In searching for new compounds, the focus may be on plant-origin molluscicides and possibly toxins from cyanobacteria. If systemic toxins with molluscicidal effect were found in cyanobacteria and if their effect was retained in dead cells, these might be ideal for inclusion in bait formulations.

Since snails are attracted to certain chemicals, these could be used to develop highly specific controlled-release formulations for the selective removal of schistosome intermediate hosts [151]. This conceptually simple model involves using an ingestible microcapsule of the optimum size and composition to release specific snail attractants, arrestants, and phagostimulants into the external environment and a toxicant in the gut of the target snail following ingestion [132]. The release of the toxicant in the gut would avoid the problem of the toxicant being a repellent. Given the ability of such formulations to both attract snails and induce ingestion, it should be possible to use them in small amounts and this should make them cost-effective [141].

To produce a viable third-stage control release formulation, a number of secondary objectives must first be achieved [132]. These include the identification of suitable bioactive factors, toxins, and formulations of the right size and chemical characteristics. Active compounds can be derived from known sources of attractants and arrestants such as plant food organisms, conspecifics, mucus trails, and conditioned media by means of the appropriate biochemical methods. Moreover, classes of chemicals known to be released exogenously by organisms which are attractive to snails can be investigated systematically to elucidate structure–activity relationships. Amino acids are included in this category as it is known that they are released exogenously by aquatic plants, which may serve as food organisms as well as by snails [151].

## 7. Conclusions and Future Outlook for Use of Molluscicides

Many schistosomiasis control programmes have been successful in reducing both disease infection levels and schistosome-caused morbidity, and while chemotherapy of infection in humans has been a major intervention, several of these have included snail control through various means [152,153,154,155].

The extensive treatment of human schistosome infection by praziquantel has been an effective public health strategy in controlling urogenital and intestinal schistosomiasis in several endemic countries [18,156]. Mass treatment with praziquantel acts only on the transmission pathway from humans to snails and only for as long as treatment is given [1]. By combining chemotherapy with known and effective control measures, such as the use of molluscicides, environmental modification, improved water supply and sanitation, and health education, multifaceted integrated control programmes can target both transmission pathways [1]. Gray et al. [1] argue that because of the focal nature of schistosome transmission, the multifaceted integrated control programme should build on historical and current data and use case-finding, geographical information systems, as well as remote sensing to develop predictive maps that would allow endemic areas and re-emerging pockets of transmission to be targeted. The approach would be tailored to specific endemic settings and be incorporated into national and local health services [1].

Molluscicide application has remained the intervention of choice for snail control despite the following problems: high costs, which may cause foreign exchange problems; lack of specificity, resulting in harm being done to fish populations, potential non-native biocontrol agents, domestic animals, and humans; strong environmental resistance due to inactivation by inorganic or organic ligands; accumulation in plants and biodegradation by bacteria; and the high costs of the development of new molluscicide products.

While there undoubtedly is a need for the development of new effective, inexpensive, and environmentally friendly molluscicides, more focus should also be devoted to developing new formulations of existing compounds. There have been some studies conducted on controlled release and bait formulations; however, there should be more focus on such formulations; especially, the ideas of environmental “antibodies” for selective removal of intermediate hosts [132] deserve further study. In China, some new niclosamide formulations have been screened and evaluated for molluscicidal activity [137]. Moreover, the mode of application of existing molluscicides might be improved. Since schistosome transmission is generally focal rather than widespread, the application of molluscicides should be restricted to such focal transmission sites to limit the use of molluscicides and their environmental impact. Based on mathematical modelling, Woolhouse et al. [157,158] found that the focal method would not be effective unless all transmission sites were treated. The use of focal mollusciciding has been performed in Saint Lucia [159,160], Sudan [31,112,161], Mali [48,162], and Egypt [49]. Focal snail control in streams in Machakos, Kenya, was shown to reduce the incidence of *S. mansoni* infection in communities depending on this stream for domestic purposes [163].

Molluscicides might be a robust tool to deploy against emerging infections, especially when these are focal, such as in Corsica (France), where the first cases of autochthonous schistosomiasis (*S. haematobium*) were very recently found [164,165]. Such newly focal transmissions need rapid and focal control with molluscicides in order not to spray in other places in France and Europe.

### 7.1. Interest in Travel Medicine and Mollusciciding around Touristic Sites and in Urban Areas

With increased tourism, many travelers may return home from travels in schistosomiasis endemic areas and doctors in their home country may not think of schistosomiasis as the cause of disease symptoms. This infection of tourists could negatively impact tourism in the endemic countries [166,167]. The most well-known example is Lake Malawi, where many tourists become infected each year [168]. In some areas, mollusciciding in sites where tourists would normally contact water might be a realistic way of protecting tourists, but not everywhere (e.g., Lake Malawi). Guidelines for tourists on how to minimize the risk of infection at Lake Malawi have been published [169].

Urban transmission of schistosomiasis has long been recognized as a problem, but with increasing rural–urban migration especially in low- and middle-income countries, and with subsequent rapid and unplanned urbanization, the problem of urban transmission has increased [170]. Due to high population density and poor living conditions, human–water contact can be very intense in natural waterbodies within the urban area [30]. Under such conditions, mollusciciding might prove efficient in reducing transmission.

### 7.2. Cost of Chemical Control and Research Need

Generally mollusciciding is costly, not just because of the price of the chemicals, but also because of the logistics and operational set-up and requirements to be sustained when transmission intensity reaches low levels. However, cost-effectiveness studies have shown that the inclusion of snail control activities is essential for optimal disease control, particularly in high prevalence areas, hot spots, and in case of non-compliance to mass drug administration when the infection prevalence becomes low [171]. Furthermore, a study comparing the cost of schistosomiasis control strategies in Zanzibar showed that mollusciding is less expensive than other interventions when not accounting for the cost of the molluscicides [172].

Therefore, there is a need to research alternative formulations of molluscicides or alternative modes of application. Molluscicides should not be applied on a wide scale because of their potential environmental hazards and effects on the other aquatic fauna; rather, focal application is preferred in transmission sites at strategic time points during the year. These time points may not be fixed from year to year, wherefore careful surveillance of the transmission situation at a given location and time must form the basis for the decision on whether to apply molluscicide. The introduction of eDNA methodologies may offer fast and efficient ways of checking for the presence of snails at potential transmission sites in addition to physical searching [173].

Mollusciciding is a realistic approach to reduce schistosomiasis transmission in small habitats, while in large habitats such as irrigation schemes and natural lakes, it may not be sustainable due to the costs and potential ecological effects. Irrigation schemes are managed habitats, and it may be more cost-effective and sustainable to implement alternatives for snail control. These could be operational procedures that might reduce disease transmission, improve water supply and sanitation, etc.

## Figures and Tables

**Table 1:** Classification of mollusciciding strategies for aquatic snails (from Appleton, 1996) [43].

Strategy	Description
**Flowing watercourses: natural streams, canals, and drains in irrigation schemes**	
Blanket spraying	covers the entire surface of a habitat, working upstream to ensure the formation of a plug of treated water that will move downstream.
Partial treatment	avoids the expense and wastage of blanket spraying, treating only a swathe ± 6–8 m wide or to a depth of 3 m, whichever is reached first, along all or part of each bank.
Controlled spillage	further reduces costs, allowing water from a treated dam to flow into target furrows after the required retention time.
Focal/contact point treatment	concentrates blanket or partial treatment at human contact points and for an arbitrary distance upstream and downstream.
Dam-and-flush treatment	impounds water in a watercourse at a bridge, treating the impounded water with the concentration needed for both the impounded volume plus that of any residual pools and tributaries downstream. Release occurs after allowing the treated water to stand for 2 h.
Drip-feed dispensing	molluscicide is fed at a pre-determined rate into flowing systems via 200 L constant head dispensers for a 12 h period once or twice per year, depending on the observed rate of recolonization by snails.
**Lentic waterbodies: farm storage dams and pools in rivers**	
Total volume treatment	the cost-effective use of this method depends on the calculation of the volume of water to be treated. Best suited to small waterbodies (<75,000 m^3^) where transmission potential is high. In larger waterbodies where potential is low, partial treatment may suffice.
Focal/contact point treatment	see above.
Slow-release formulations	molluscicide-impregnated rubber or glass matrices are scattered on the substratum at a given density. Snail control depends on chronic intoxication after a lag period.

## Data Availability

Not applicable.
